# Editorial: Neuroplasticity and Extracellular Proteolysis

**DOI:** 10.3389/fncel.2016.00059

**Published:** 2016-03-11

**Authors:** Jerzy W. Mozrzymas, Leszek Kaczmarek

**Affiliations:** ^1^Laboratory of Neuroscience, Department of Biophysics, Wroclaw Medical UniversityWroclaw, Poland; ^2^Molecular and Cellular Neurobiology, Nencki Institute of Experimental BiologyWarsaw, Poland

**Keywords:** metalloproteinases, proteolysis, neuroplasticity, extracellular matrix, learning and memory

For long investigations of neuroplasticity and research into the role of extracellular proteolysis have been progressing largely separately but their recent progress was paralleled by a growing awareness that neuronal networks form a functional partnership with glia and extracellular matrix (ECM) and thus a term “tetrapartite synapse” was coined (Dityatev and Rusakov). Extracellular proteolysis emerges now as a key mechanism in mediating interactions between these players and description of this cross-talk opens new avenues in exploring physiological and pathological mechanisms in the CNS. The present research topic aims at presenting some different facets of proteolysis in a broad context of neuroplasticity.

Reinhard et al. provide an overview on MMP-9 at the levels and regulation of enzymatic activity, protein, and mRNA, in the brain development with emphasis on critical periods in the sensory cortices. Important role of the enzyme in neuronal/synaptic plasticity is presented along pointing to gaps in this field. Notably, new results are provided by Kelly et al. that loss of MMP-9 attenuates functional ocular dominance and reduces excitatory synapse density in the visual cortex. Kelly et al. observed no change in the morphology of existing dendritic spines, however, spine dynamics were altered in MMP-9 KO mice. Reinhard et al. further discuss cellular and molecular mechanisms in which MMP-9 plays paramount role to control synapse development and plasticity. The authors review studies on long-term potentiation (LTP) to conclude on essential role of MMP-9 in LTP maintenance, as opposed to its early phase. Interestingly, Gorkiewicz et al. demonstrate that MMP-9 is pivotal for the lasting LTP evoked on the lateral to basal as well as basal to central amygdala connections but not in the cortical to lateral amygdala pathway. Mechanisms of plastic changes may differ between projections raising a possibility of different involvement of metalloproteinases. Wiera and Mozrzymas review involvement of metalloproteinases in mossy fiber synapses onto CA3 pyramidal cells (MF-CA3) projection that show pronounced short-term facilitation, a pre-synaptic and NMDAR-independent LTP and a prominent structural plasticity induced by learning. Reinhard et al. discuss also MMP effects on glutamate receptors trafficking and morphology of the dendritic spines. Excessive MMP-9 provokes dendritic spine thinning and elongation, whereas when MMP-9 activity is counterbalanced by its endogenous inhibitors, the enzyme contributes to the spine head expansion. At the molecular level, MMP-9 function at the spines appears to be mediated by cleavage of cell adhesion molecules (CAM) and integrin signaling. This issue has been reviewed by Conant et al. They discuss the role of a variety of metalloproteinases in the context of LTP and learning and memory with particular emphasis of CAM cleavage which may provide means to generate integrin-binding ligands.

Besides synaptic plasticity, neuronal excitability may undergo plastic changes affecting thus the output of dendritic integration. Wójtowicz et al. discuss role of metalloproteinases in this form of plasticity. Ben Shimon et al. provide an extensive review of the role of thrombin which, through direct or indirect activation of Protease-Activated Receptor-1 exerts e variety of effects on classic (LTP) and homeostatic synaptic plasticity and was shown to be involved in epileptogenesis.

The MMP-9 targets are addressed by Kelly et al., who show that critical period for visual cortex development coincides with massive degradation of chondroitin sulfate proteoglycans that is prevented in MMP-9 KO mice. Further, molecule functionally linking MMPs with ECM is CD44, whose molecular interactions, signaling and roles in the nervous system are reviewed by Dzwonek et al. They show great functional diversity of CD44 that is important in a wide range of physiological and pathological phenomena, e.g., axon guidance, cytoplasmic calcium clearance, dendritic arborization, synaptic transmission, epileptogenesis, oligodendrocyte, and astrocyte differentiation, post-traumatic brain repair, and brain tumor development. Bijata et al. add an important piece of information that MMP-9 contributes to dendritic development in hippocampal neurons in vitro and provide evidence that MMP-9 mediated cleavage of beta-dystroglycan is important for dendritic development.

As discussed by Reinhard et al., MMP-9 dysfunctions contribute to major neuropsychiatric pathologies, such as Fraxile X Syndrome (FXS) and other forms of autism spectrum disorders, bipolar disorder, schizophrenia, and epilepsy that may all share neurodevelopmental provenance. Therapeutic approaches aiming at MMP-9 inhibition by non-specific drugs such as doxycycline and minocycline have already been shown to be beneficial also in humans suffering from FXS. The role of MMP-9 in FXS is also discussed by Conant et al., who extend the review of MMP-dependent animal models of psychiatric conditions also to addiction.

GABAergic interneurons (particularly parvalbumin-positive) play a pivotal role in shaping network rhythmicity, which is crucial in cognition. Suzuki et al. examine the correlation between neuropsin and parvalbumin (PV) expression in a model of mice freely moving in a familiar or enriched environment. Whereas, neuropsin knockout resulted in reduction of PV reactivity in mice reared in the familiar environment, in the enriched one, upregulation of PV expression was observed in both groups.

A thorough description of ECM composition is a pre-requisite for exploring the mechanisms whereby proteolysis shapes the neuroplasticity. Malik et al. describe the matricellular proteins of the CCN family which was implicated in regulation of e.g., gene expression, proliferation, differentiation, adhesion, and migration. CCN proteins have been studied mostly beyond CNS, however there is neuronal expression of CCN proteins and their contribution to nervous system development, function, and pathology should be considered.

In aggregate, the papers published in this volume rather open new avenues of research than sum up a scientific field close to completion. Upregulation of extracellular proteases has long been believed to accompany mainly pathological conditions. Extensive research over past two decades has provided strong evidence that these enzymes play pivotal roles in physiological and aberrant synaptic plasticity, as shown in this volume. It is up for the further studies to verify a claim that extracellular proteolysis comes of age, holding an important key to understand brain function, and dysfunction; a key that can be also used to develop diagnostic methods and therapies to combat the neuropsychiatric disorders.

## Author contributions

All authors listed, have made substantial, direct and intellectual contribution to the work, and approved it for publication.

## Funding

This work has been supported by grant of National Centre of Science to JWM, grant number: DEC-2013/11/B/NZ3/00983.

### Conflict of interest statement

The authors declare that the research was conducted in the absence of any commercial or financial relationships that could be construed as a potential conflict of interest.

